# Precision MRI phenotyping of muscle volume and quality at a population scale

**DOI:** 10.3389/fphys.2024.1288657

**Published:** 2024-02-02

**Authors:** Marjola Thanaj, Nicolas Basty, Brandon Whitcher, Elena P. Sorokin, Yi Liu, Ramprakash Srinivasan, Madeleine Cule, E. Louise Thomas, Jimmy D. Bell

**Affiliations:** ^1^ Research Centre for Optimal Health, School of Life Sciences, University of Westminster, London, United Kingdom; ^2^ Calico Life Sciences LLC, South San Francisco, CA, United States

**Keywords:** muscle volume, muscle quality, intermuscular adipose tissue, magnetic resonance imaging, dynapenia, frailty

## Abstract

**Introduction:** Magnetic resonance imaging (MRI) enables direct measurements of muscle volume and quality, allowing for an in-depth understanding of their associations with anthropometric traits, and health conditions. However, it is unclear which muscle volume measurements: total muscle volume, regional measurements, measurements of muscle quality: intermuscular adipose tissue (IMAT) or proton density fat fraction (PDFF), are most informative and associate with relevant health conditions such as dynapenia and frailty.

**Methods:** We have measured image-derived phenotypes (IDPs) including total and regional muscle volumes and measures of muscle quality, derived from the neck-to-knee Dixon images in 44,520 UK Biobank participants. We further segmented paraspinal muscle from 2D quantitative MRI to quantify muscle PDFF and iron concentration. We defined dynapenia based on grip strength below sex-specific cut-off points and frailty based on five criteria (weight loss, exhaustion, grip strength, low physical activity and slow walking pace). We used logistic regression to investigate the association between muscle volume and quality measurements and dynapenia and frailty.

**Results:** Muscle volumes were significantly higher in male compared with female participants, even after correcting for height while, IMAT (corrected for muscle volume) and paraspinal muscle PDFF were significantly higher in female compared with male participants. From the overall cohort, 7.6% (N = 3,261) were identified with dynapenia, and 1.1% (N = 455) with frailty. Dynapenia and frailty were positively associated with age and negatively associated with physical activity levels. Additionally, reduced muscle volume and quality measurements were associated with both dynapenia and frailty. In dynapenia, muscle volume IDPs were most informative, particularly total muscle exhibiting odds ratios (OR) of 0.392, while for frailty, muscle quality was found to be most informative, in particular thigh IMAT volume indexed to height squared (OR = 1.396), both with *p*-values below the Bonferroni-corrected threshold ( $p<8.8{\times 10}^{-5}$ ).

**Conclusion:** Our fully automated method enables the quantification of muscle volumes and quality suitable for large population-based studies. For dynapenia, muscle volumes particularly those including greater body coverage such as total muscle are the most informative, whilst, for frailty, markers of muscle quality were the most informative IDPs. These results suggest that different measurements may have varying diagnostic values for different health conditions.

## 1 Introduction

Direct population-scale measurements of muscle have been relatively limited, most studies have used measures of fat-free mass (FFM) by bioelectrical impedance analysis (BIA), or measures of lean body mass (LBM) from dual-energy X-ray absorptiometry (DXA) (Hofsteenge et al., 2015; Zillikens et al., 2017). Unlike direct muscle measures, FFM contains all nonfat components of the body, while LBM additionally contains significant and disparate contributions from organs, skin, bones, body water, and essential fat (Scafoglieri and Clarys, 2018). On the other hand, magnetic resonance imaging (MRI) enables the direct quantification of both muscle mass and quality, with the latter, generally related to levels of muscle fat infiltration (Gallagher et al., 2005; Linge et al., 2018).

Until recently, measurement of total body muscle volume and distribution by MRI has been typically limited to relatively small cohorts, due to the cost and time-consuming requirements of image acquisition and analysis. The lack of automated techniques led researchers to rely on the measurement of single or multiple cross-sectional areas as an index of overall muscle mass, with a single slice at the third lumbar vertebra (L3) (Schweitzer et al., 2015), individual muscles groups such as the iliopsoas (Fitzpatrick et al., 2020) or anatomical groupings such as the. thigh muscles (Linge et al., 2018) being among the most popular approaches. The extent to which proxies of muscle volume can provide sufficient information to discern the overall impact of muscle volume and quality on health is yet to be fully ascertained. Additionally, there is a lack of consensus regarding whether muscle measurements should be used independently, as ratios or whether these should be indexed to height (Linge et al., 2020).

Ageing and morbidity-related declines in physical function have shown to be associated with a loss of muscle mass, strength and reduction in muscle quality (Ungar and Marchionni, 2017; Sizoo et al., 2021). While muscle-related conditions such as sarcopenia and frailty are typically associated with ageing, it is increasingly apparent that multiple long-term factors increase the risk of these conditions at a relatively younger age (Hanlon et al., 2018; Dodds et al., 2020). Despite their common association with ageing and multiple diseases, previous studies have shown that even low muscle strength, termed as dynapenia (measured by hand grip strength) is associated with hallmarks of ageing (Jones et al., 2021) and is predictive of future long-term morbidity and mortality (Rantanen et al., 1999; Mitchell et al., 2012).

Recent MRI studies have shown the combination of low muscle mass and high levels of muscle fat infiltration to be a predictor of all-cause mortality, suggesting that muscle fat infiltration could contribute to the definition of sarcopenia (Linge et al., 2021). However, much of this work has been limited to specific anatomical regions such as the thigh muscle. Additionally, there is a growing interest in measuring the size of the iliopsoas muscle, primarily due to its potential as a marker of sarcopenia. Indeed, it has been suggested that this muscle group appears to indirectly influence conditions associated with sarcopenia and frailty, including adverse health outcomes such as morbidity and mortality (Ebbeling et al., 2014; Kasahara et al., 2017). Furthermore, previous studies have shown that atrophy and changes in fat in paraspinal muscles, stemming from sarcopenia, are linked to functional impairments and chronic back pain (Masaki et al., 2016; Kim et al., 2019), making these muscles important subjects of study in the context of ageing and health.

While the UK Biobank imaging protocol does not encompass the entire body, including only the neck-to-knee region, it is still possible to derive meaningful overall muscle measures including overall muscle volume within that region, specific muscle groups including iliopsoas muscle, thigh muscles, paraspinal muscles, as well as intermuscular adipose tissue (IMAT). In this study, we report both MRI muscle mass and quality measurements for 44,520 UK Biobank participants. We define our muscle volume image-derived phenotypes (IDPs) as total muscle volume, iliopsoas muscle volume, thigh and mid-thigh muscle volume. Additionally, our muscle quality IDPs include thigh IMAT volume, mid-thigh IMAT volume, fat in the paraspinal muscles and the ratio of the mid-thigh IMAT to muscle volume. We further assess which IDPs related to muscle volume and quality, both in their unadjusted form and after adjusting for the most common allometric scaling (height squared), are most informative and associate them with anthropometric factors and relevant health conditions including dynapenia and frailty.

## 2 Materials and methods

### 2.1 Data

A total of 44,520 UK Biobank participants were included in this analysis. Participant data from the cohort was obtained through UK Biobank Access Application number 44584. The UK Biobank has approval from the North West Multi-Centre Research Ethics Committee (REC reference: 11/NW/0382). All measurements were obtained under these ethics, adhering to relevant guidelines and regulations, with written informed consent obtained from all participants. Researchers may apply to use the UK Biobank data resources and the results generated in this study, by submitting a health-related research proposal that is in the public interest. More information may be found on the UK Biobank researchers and resource catalogue pages (https://www.ukbiobank.ac.uk).

### 2.2 Image processing and MRI measurements

Full details of the UK Biobank magnetic resonance imaging (MRI) abdominal protocol have previously been reported (Littlejohns et al., 2020). The data presented in this paper primarily consisted of two MRI acquisition methods: the chemical-shift-based water-fat separation MRI, commonly known as Dixon, which covered the region from the neck to the knee, and a distinct single-slice multiecho MRI acquisition specific to the liver. All data were analysed using our dedicated image processing pipelines for the Dixon and two types of single-slice multiecho acquisitions: (i) Iterative Decomposition of Water and Fat with Echo Asymmetry and Least-Squares Estimation (IDEAL) and (ii) gradient echo (GRE), with deep learning algorithms employed to segment organs and tissue (Liu et al., 2021). Proton density fat fraction (PDFF) and R2* were calculated from the Phase Regularized Estimation using Smoothing and Constrained Optimization (PRESCO) method from both the single-slice IDEAL and GRE acquisitions (Liu et al., 2021).

Our pipeline generates more than 30 image-derived phenotypes (IDPs) from the Dixon MRI data, the subset of IDPs included in this study are primarily muscle volumes, including *total muscle* (within neck-to-knee volume), *thigh (left + right) muscle volume and iliopsoas (left + right) muscle volume*. For training the deep learning model, we utilised 108 manual annotations of total muscle and 151 annotations of the iliopsoas muscles. The Dice similarity coefficients on 20% out-of-sample testing data were 0.94 for total muscle and 0.95 for the iliopsoas muscle segmentations. The thigh muscle volumes were derived from the total muscle segmentations using anatomical landmarks from the femur. Together with these IDPs, to avoid any bias of height we also analysed a 10 cm image slab, placed at the midpoint of the length of the femur on each leg. This is referred to as the mid-thigh region throughout the text.

In addition to muscle volume IDPs several adipose tissue depots were extracted, including abdominal subcutaneous adipose tissue (ASAT), visceral adipose tissue (VAT) and intermuscular adipose tissue (IMAT, the tissue located between muscle groups) volumes (Liu et al., 2021). We also obtained a measure of intramyocellular fat stored in the paraspinal muscles, referred to as PDFF to avoid confusion. We finally extracted a measure of paraspinal muscle iron concentration by transforming the R2* to iron concentration in mg/g (Wood et al., 2005). For model training, we had 195 manual annotations of the IDEAL paraspinal muscles and 187 manual annotations of the GRE paraspinal muscles. The Dice similarity coefficient on 20% out-of-sample testing data was 0.81 and 0.86 for the paraspinal muscle segmentations in IDEAL and GRE acquisitions, respectively. All annotations were carefully selected to be representative of the UK biobank population, covering a broad range of anthropometric characteristics and conditions such as dynapenia and frailty reflecting the expected ranges of fat and muscle content. This approach was designed to ensure the generalisability of our pipeline, and the segmentation quality was further confirmed through visual inspection at multiple stages by experienced analysts before use in the segmentation, as described in (Liu et al., 2021).

To account for the potential confounding effect of height on muscle and fat volumes, IDPs were indexed to the commonly used allometric scaling, height squared (Linge et al., 2020). Muscle volume was defined for all muscle IDPs including total muscle volume, iliopsoas muscle volume, thigh and mid-thigh muscle volume, whereas muscle quality was defined from all fat measurements including thigh IMAT volume, mid-thigh IMAT volume and paraspinal muscle PDFF. We further evaluate muscle quality measurement related to IMAT, correcting for muscle volume in the mid-thigh. This correction is represented as the mid-thigh IMAT/muscle ratio calculated as follows: (mid-thigh IMAT volume/(mid-thigh IMAT volume + mid-thigh muscle volume)) * 100. Based on the known association between levels of muscle fat infiltration (referred to as IMAT) and muscle function (Linge et al., 2020), a participants quality of muscle is reflected by their IMAT levels. Hence, increased IMAT corresponds to reduced muscle quality. Details regarding MR acquisition parameters are as previously described (Liu et al., 2021). All relevant data and associated materials, including MR acquisition parameters and deep learning architecture, will be made available to the public according to the well-established UK Biobank protocol.

### 2.3 Quality control

IDPs with insufficient anatomical coverage were reported as missing values and a visual inspection of the MRI data was performed to determine potential extreme values in the IDPs to confirm exclusion. To ensure the full anatomical coverage of the organs a threshold was defined as follows:

• *Total muscle volume*: 5 L < total muscle <60 L. No participants were identified above or below this threshold.
• *Thigh muscle volume*: 1 L < thigh muscle <15 L. A total of 25 participants were identified and removed from all subsequent analyses.
• *Thigh IMAT volume*: thigh IMAT >50 mL. One participant was identified and removed from all subsequent analyses.
• Images from all excluded participants were subsequently visually inspected to confirm the original finding.
• A total of 87 participants were excluded from the volumetric analysis of the Dixon MRI data due to missing values. We excluded 1,547 participants for the muscle and fat volume index analyses, mainly due to missing standing height data. From the single-slice multiecho data 319 participants were excluded from the PDFF and iron concentration analysis in the paraspinal muscle due to missing values.

### 2.4 Phenotype definitions

Anthropometric measurements including age, body mass index (BMI), waist and hip circumferences and hand grip strength (HGS) were taken at the UK Biobank imaging visit. Ethnicity was self-reported at the initial assessment visit and was categorised as follows: White (any British, Irish or other white background); Asian (any Indian, Pakistani, Bangladeshi or any other South Asian background); Black (any Caribbean, African or any other Black background); Chinese and Others (mixed ethnic background including White and Black Caribbean, White and Black African, White and Asian, and other ethnic groups). Sex was self-reported and included those recorded by the NHS and those obtained at the initial assessment visit. The Townsend deprivation index was calculated immediately prior to participants joining the UK Biobank.

Participants excess metabolic equivalents of task (MET) in hours per week were computed by multiplying the time in minutes spent in each activity, including walking, moderate and vigorous activity, on a typical day by the number of reported days doing the exercise and the respective MET scores using the methods previously described (ODonnell et al., 2020). Excess METs scores were at 2.3, 3 and 7 for walking, moderate and vigorous activity, respectively (ODonnell et al., 2020). The total physical activity MET was computed by taking the sum of walking, moderate and vigorous MET in hours per week. Any activity lasting less than 10 min on a typical day was recorded to 0 for any of the three categories of activity (walking, moderate and vigorous activity). Additionally, for each of the three categories of activity, values exceeding 1,260 min per week (equivalent to an average of 3 h per day) were truncated at 1,260 min (Bradbury et al., 2017). In our following analysis total MET will be referred to as MET. All physical activity measures, alcohol intake frequency and smoking status were self-reported at the UK Biobank imaging visit.

Relevant conditions of interest including dynapenia and frailty were defined according to published criteria:

• *Dynapenia* was defined as low muscle strength with HGS below 27 kg in male participants and below 16 kg in female participants (Dodds et al., 2014; 2020; Silva et al., 2022; Wilkinson et al., 2022). The HGS used was that of the dominant hand. If participants reported using both hands, the mean of the right and left hand was utilised. Initially, we planned a stricter definition of sarcopenia including low muscle quality as recommended by EWGSOP2 (Cruz-Jentoft et al., 2019) which combines both HGS and DXA-measured appendicular lean mass (ALM)/height^2^: 6.0/7.0 kg/m^2^ (female/male participants). However, due to the limited availability of UKBB DXA data, (only 4,683 participants had available DXA measurements) resulting in only 0.3% (78M/22F) of the cohort meeting the full sarcopenia criteria. Hence, our analysis was focused only on dynapenia.
• *Frailty* was defined using the criteria adopted by (Hanlon et al., 2018), to be used with self-reported UK Biobank questionnaire responses which required the presence of 3 out of 5 indicators. These indicators included weight loss, exhaustion (more than half the days or nearly every day), no or only light (once a week or less) physical activity in the last 4 weeks, slow walking speed, and low HGS (Supplementary Table S1). All the frailty indicators were taken at the UK Biobank imaging visit. Additional classifications included pre-frailty (1 or 2 of the 5 indicators), and not frail (none of the indicators).

### 2.5 Statistical analysis

All summary statistics, hypothesis tests, regression models and figures were performed in R software environment version 4.1.3. Figures were produced using the *ggplot2* package (Wilkinson, 2011). All descriptive characteristics are presented as means with standard deviations (SD) for quantitative variables and as percentages for categorical variables. Spearmans rank correlation coefficient (ρ) was used to assess monotonic trends between variables. The Wilcoxon rank-sum test was used to compare the means between two groups. One-way ANOVA was used to compare multiple groups.

We explored the association between muscle and fat IDPs and dynapenia by performing a logistic regression analysis model adjusting for age, sex, ethnicity, alcohol intake frequency, smoking status, Townsend deprivation index, MET and the muscle and fat IDPs including total muscle volume, thigh muscle volume, mid-thigh muscle volume, iliopsoas muscle volume, thigh IMAT volume, mid-thigh IMAT volume, mid-thigh IMAT/muscle volume and paraspinal muscle PDFF. Additionally, we incorporated paraspinal muscle iron concentration in our model due to its established association with adiposity and metabolic-related conditions (Moreno-Navarrete et al., 2016) We further applied an ordinal logistic regression model to investigate the association between muscle and fat IDPs on frailty given it has multiple ordered categories (not frail, pre-frail, and frail). Ordinal logistic regression models were adjusted for all the variables used in the above logistic regression models. The ordinal logistic regression models were performed using the R *MASS* package (Venables and Ripley, 2013). Because muscle and fat IDPs are associated with body composition measurements such as waist-to-hip ratio and BMI, adjustment for these categories might result in over-adjustment bias (Schistermanet al., 2009). Furthermore, HGS was excluded from logistic models since it is used in the definition of dynapenia. In all models iliopsoas muscle, thigh muscle, thigh IMAT volumes, and indices are shown as the sum of left and right volumes). All anthropometric variables and IDPs were standardised prior to inclusion in the logistic regression models. Thus, we will refer to these models as standardised logistic regression models.

Summaries of the standardised logistic regression model are reported as odds ratios (OR) with 95% confidence intervals (CIs). To assess the performance of each standardised logistic regression model we used the following metrics: Akaike Information Criterion (AIC), the area under the curve (AUC) of the receiver operating characteristic (ROC) with corresponding CIs for dynapenia and concordance index (c-index) for frailty. Lower AIC values indicate a better model fit, while higher AUC and c-index values suggest better discrimination between cases. AUC was calculated using the *pROC* R package (Robin et al., 2011), while the c-index was computed using the *rms* R package (Harrell, 2013). The Bonferroni-corrected threshold for statistical significance was $0.05/568=8.8\times 10^{-5}$ .

## 3 Results

**FIGURE 1:**
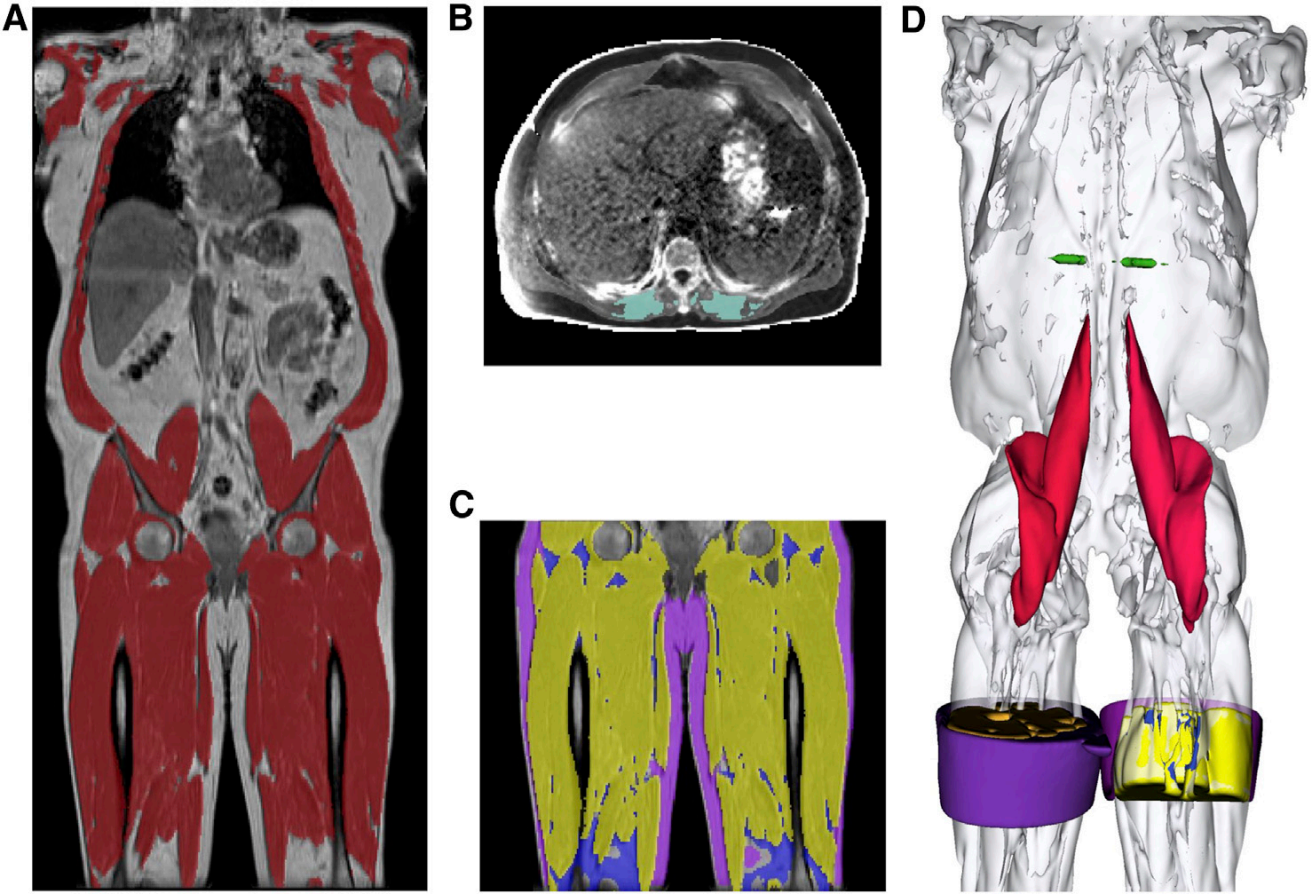
Segmentations from the UK Biobank abdominal MRI of a participant showing: **(A)** the total muscle (dark red) overlaid on the coronal view of a neck-to-knee scan, **(B)** the paraspinal muscle (green) in 2D quantitative MRI, **(C)** the thigh muscles (yellow), thigh IMAT (blue), thigh SAT (purple) overlaid on the coronal view, and **(D)** 3D renderings of the muscle segmentations for the following structures: mid-thigh muscle (yellow), mid-thigh IMAT (blue), mid-thigh subcutaneous adipose tissue (purple), paraspinal muscles (green), iliopsoas muscles (red) and total muscle (white).

Typical images showing muscle and fat IDPs generated from each participant are shown in Figure 1. Image datasets were available from 44,520 individuals, with all required IDPs being successfully derived for each participant.

### 3.1 Study population characteristics

Summary statistics for the study population are provided in Table 1, 51.7% were female participants with similar average age to male (female participants 63.54 ± 7.58 years and male participants 64.93 ± 7.83 years). The average BMI for female and male participants were 26.02 ± 4.72 kg/m^2^ (range 13.4–62.0 kg/m^2^) and 26.95 ± 3.90 kg/m^2^ (range 16.4–58.1 kg/m^2^) respectively. HGS in the dominant hand was 38.82 ± 8.85 kg in male participants and 23.98 ± 6.12 kg in female participants and the MET was reported as 24.03 ± 17.51 hours/week from female participants and 25.16 ± 17.47 hours/week from male participants (Table 1). The overall ethnic distribution for the cohort was 96.7% White, 1.1% Asian, 0.7% Black, 0.3% Chinese and 1% Others (see methods for details).

**TABLE 1:** Demographics of the participants (N = 44,520), separated by sex.

	Full cohort	Female	Male
N	44,520	23,013	21,507
White (N)	43,059	22,277	20,782
Asian (N)	476	175	301
Black (N)	297	164	133
Chinese (N)	133	80	53
Others (N)	438	265	173
Age (yrs.)	64.21 ± 7.73	63.54 ± 7.58	64.93 ± 7.83
Weight (kg)	75.99 ± 15.10	68.88 ± 13.03	83.60 ± 13.35
Height (cm)	169.17 ± 9.27	162.72 ± 6.25	176.06 ± 6.64
BMI (kg/m^2^)	26.47 ± 4.37	26.02 ± 4.72	26.95 ± 3.90
Waist Circumference (cm)	88.37 ± 12.69	82.78 ± 11.83	94.34 ± 10.71
Hip Circumference (cm)	100.76 ± 8.71	100.81 ± 9.83	100.70 ± 7.34
Waist-to-Hip Ratio	0.88 ± 0.09	0.82 ± 0.07	0.94 ± 0.06
Dominant HGS (kg)	31.17 ± 10.60	23.98 ± 6.12	38.82 ± 8.85
Walking MET (hours/week)	9.16 ± 7.23	9.18 ± 7.33	9.15 ± 7.12
Moderate MET (hours/week)	8.55 ± 7.81	8.61 ± 7.88	8.49 ± 7.74
Vigorous MET (hours/week)	6.86 ± 7.94	6.25 ± 7.68	7.52 ± 8.16
Total MET (hours/week)	24.58 ± 17.49	24.03 ± 17.51	25.16 ± 17.47
Townsend deprivation index	−1.88 ± 2.73	−1.83 ± 2.74	−1.93 ± 2.72
Alcohol frequency (N)			
Daily	7,585	3,118	4,467
1–4 times per week	24,014	11,802	12,212
1–3 times per month	5,130	3,081	2,049
Occasionally or never	7,469	4,841	2,628
Smoking status (N)			
Never	27,481	15,035	12,446
Previous	15,067	7,068	7,999
Current	1,518	657	861
VAT (L)	3.95 ± 2.31	2.82 ± 1.57	5.16 ± 2.36
ASAT (L)	8.47 ± 4.12	9.82 ± 4.37	7.01 ± 3.27

Values are reported as mean and standard deviation for continuous variables and counts (N) for categorical variables. BMI, Body mass index; HGS, Hand grip strength; MET, Metabolic equivalents of task; VAT, Visceral adipose tissue; ASAT, Abdominal subcutaneous adipose tissue.

### 3.2 Muscle volume and quality characteristics

Total muscle in female and male participants were 14.04 ± 1.95 L and 21.80 ± 3.00 L (*p* < $8.8\times 10^{-5}$ ) respectively, with a mean difference between the sexes of 7.76 ± 3.60 L, or 43% (Table 2). Similar proportional sex differences were observed for all other muscle IDPs. We also observed significant differences between left and right muscle volumes for the thigh, mid-thigh, and iliopsoas muscles in both female and male participants (Supplementary Table S2). Mid-thigh IMAT volumes, reflecting reduced muscle quality, were higher in male than female participants (55.19 ± 50.27 mL vs. 48.19 ± 38.40 mL, respectively) (Table 2). However, this trend was reversed when IMAT was corrected for muscle volume in the mid-thigh, represented as mid-thigh IMAT/muscle ratio. In this context, female participants exhibited a percentage of 2.48% ± 1.86%, whereas male participants demonstrated 1.97% ± 1.84% (*p* < $8.8\times 10^{-5}$ ) (Table 2). Paraspinal muscle PDFF, was 7.84% ± 4.03% in female and 6.88% ± 3.64% in male participants (*p* < $8.8\times 10^{-5}$ ), while iron content showed a small but significant difference between sexes (Supplementary Table S2).

**TABLE 2:** Summary statistics for muscle volume and quality IDPs.

	Full cohort (N = 44,520)	Female (N = 23,013)	Male (N = 21,507)	*p*-values
Muscle volume IDPs				
Total Muscle Volume (L)	17.79 ± 4.62	14.04 ± 1.95	21.80 ± 3.00	${\;p<10}^{-16}$
Total Muscle Volume Index (L/m^2^)	6.14 ± 1.12	5.30 ± 0.62	7.03 ± 0.81	${\;p<10}^{-16}$
Thigh Muscle Volume (L)	8.45 ± 2.23	6.68 ± 1.01	10.34 ± 1.53	${\;p<10}^{-16}$
Thigh Muscle Volume Index (L/m^2^)	2.91 ± 0.54	2.52 ± 0.31	3.33 ± 0.40	${\;p<10}^{-16}$
Mid-thigh Muscle Volume (L)	2.28 ± 0.55	1.87 ± 0.28	2.73 ± 0.41	${\;p<10}^{-16}$
Iliopsoas Muscle Volume (L)	0.64 ± 0.17	0.51 ± 0.08	0.78 ± 0.12	${\;p<10}^{-16}$
Iliopsoas Muscle Volume Index (L/m^2^)	0.22 ± 0.04	0.19 ± 0.02	0.25 ± 0.03	${\;p<10}^{-16}$
Muscle quality IDPs				
Thigh IMAT Volume (L)	0.77 ± 0.34	0.71 ± 0.29	0.84 ± 0.37	${\;p<10}^{-16}$
Thigh IMAT Volume Index (mL/m^2^)	269.82 ± 114.91	266.61 ± 109.33	273.24 ± 120.49	0.00039
Mid-thigh IMAT Volume (mL)	51.57 ± 44.67	48.19 ± 38.40	55.19 ± 50.27	${\;p<10}^{-16}$
Mid-thigh IMAT/Muscle (%)	2.24 ± 1.87	2.48 ± 1.86	1.97 ± 1.84	${\;p<10}^{-16}$
Paraspinal Muscle PDFF (%)	7.38 ± 3.88	7.84 ± 4.03	6.88 ± 3.64	${\;p<10}^{-16}$
Paraspinal Muscle Iron Concentration (mg/g)	1.20 ± 0.12	1.20 ± 0.13	1.19 ± 0.10	${\;p<10}^{-16}$

Values are reported as mean and standard deviation. Significance refers to the *p*-value for a Wilcoxon rank-sums test, where the null hypothesis is the medians between the two groups (male and female participants) being equal. IMAT: intermuscular adipose tissue; PDFF: proton density fat fraction.

### 3.3 Associations between dynapenia and frailty and muscle health

#### 3.3.1 Participant characteristics

**FIGURE 2:**
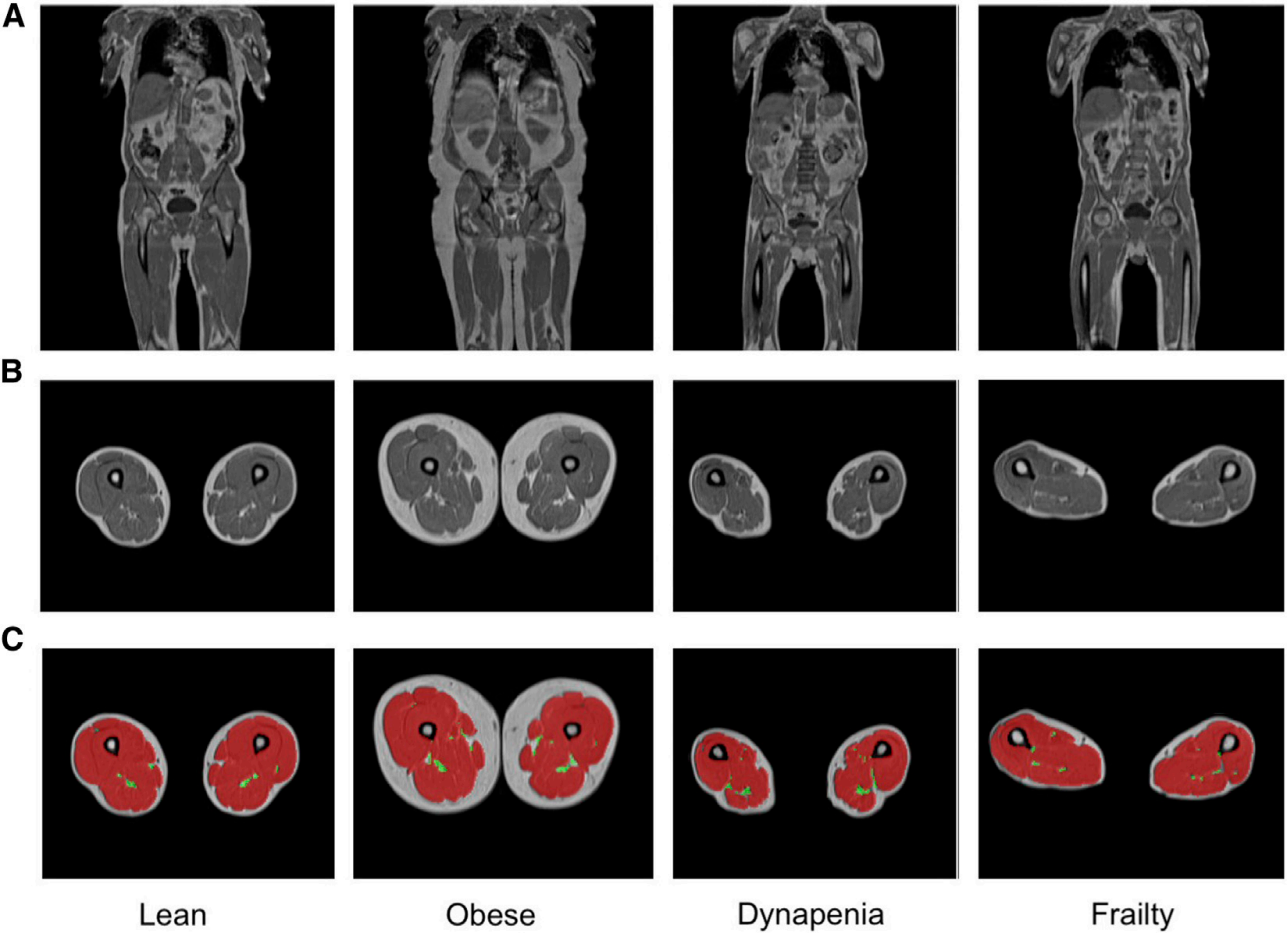
Representative samples of **(A)** coronal and **(B)** axial MRI views and **(C)** the total muscle (red) and the thigh IMAT (green) segmentations overlaid on the axial views, obtained from lean, obese, dynapenic and frail participants from the Dixon acquisition.

From the overall cohort of 44,520 participants 3,261 (7.6%) were classified as having dynapenia, and 455 (1.1%) were classified as frail (Supplementary Table S3). Representative examples of MRI images from these subgroups are shown in Figure 2. The characteristics as well as the summaries of muscle and fat IDPs of dynapenia and frailty participants are presented in Supplementary Tables S4–S7. In summary, participants with and without dynapenia were aged 67.96 ± 7.17 and 63.86 ± 7.69 years old, respectively. Not-frail, pre-frail and frail participants were 63.65 ± 7.59, 64.94 ± 7.79 and 66.56 ± 8.04 years old, respectively. Participants with dynapenia also had lower physical activity levels and a higher waist-to-hip ratio (*p* < $8.8\times 10^{-5}$ ). Similar results were observed for frailty. The average total muscle volume for participants without dynapenia was 17.92 ± 4.64 L whereas for participants with dynapenia was 16.49 ± 4.20 L (*p* < $8.8\times 10^{-5}$ ). For participants without frailty and with pre-frailty, the average total muscle volume was 17.95 ± 4.67 and 17.70 ± 4.57 L, respectively, while frail participants had an average total muscle volume of 16.59 ± 4.13 L. Additionally, muscle quality measurements such as the mid-thigh IMAT/Muscle ratio was higher in participants with dynapenia and frailty (2.19 ± 1.84 for no dynapenia; 2.71% ± 2.17% for dynapenia; 1.98 ± 1.59 for not-frail; 2.49 ± 1.93 for pre-frail and 3.73% ± 2.86% for frail). Furthermore, there was a significant correlation between dominant HGS and muscle IDPs among participants without dynapenia for both male and female participants, showing the highest correlation with total muscle volume (male participants: ρ = 0.35; female participants ρ = 0.38, both $p<8.8{\times 10}^{-5}$ ), however, this relationship lost significance in the presence of dynapenia (Supplementary Figure S1).

#### 3.3.2 Logistic regression analysis

To determine which IDPs were associated with dynapenia and frailty, we created logistic regression models, adjusting for age, sex, ethnicity, alcohol intake frequency, smoking status, Townsend deprivation index and MET, with standardisation applied to all variables. Our findings reveal that both dynapenia and frailty were positively associated with age while displaying negative associations with alcohol and MET in all models. Dynapenia displayed a positive association with male participants only in models including muscle volume IDPs, while frailty was only positively associated in the models including total, thigh and iliopsoas muscle IDPs as well as mid-thigh IMAT/Muscle ratio and paraspinal muscle PDFF (Supplementary Tables S8, S9). Additionally, smaller muscle volume IDPs and larger muscle fat IDPs were associated with the presence of dynapenia and frailty. Specifically, dynapenia and frailty were negatively associated with total muscle volume (OR = 0.392, $p<8.8{\times 10}^{-5}$ , for dynapenia; OR = 0.894, $p<8.8{\times 10}^{-5}$ , for frailty). In contrast, thigh IMAT volume index demonstrated a positive association with increased odds ratios for dynapenia (OR = 1.129, $p<8.8{\times 10}^{-5}$ ) and frailty (OR = 1.410, $p<8.8{\times 10}^{-5}$ ). Paraspinal muscle PDFF was also significantly associated with dynapenia and frailty showing increased odds ratios (OR = 1.094, $p<8.8{\times 10}^{-5}$ , for dynapenia; OR = 1.350, $p<8.8{\times 10}^{-5}$ , for frailty) while, paraspinal muscle iron concentration was not significantly associated with any of these conditions.

#### 3.3.3 Performance evaluation

Performance judged by the model with the lowest AIC (Akaikes Information Criterion) and the highest AUC and c-index (Supplementary Tables S8–S10), showed that for dynapenia, total muscle (AUC = 0.707 (0.698, 0.716 95% CI)) and total thigh volume (AUC = 0.707 (0.697, 0.716 95% CI)) were the most informative IDPs (Supplementary Tables S8, S10), with muscle quality IDPs providing the least information. Interestingly, the indexed volumes demonstrated a relatively poor fit in their associations with dynapenia. Conversely, for frailty, measures of muscle quality including thigh IMAT volume index (c-index = 0.621) and mid-thigh IMAT/Muscle ratio (c-index = 0.618) were the most informative, with muscle volumes IDPs providing less information (Supplementary Tables S9, S10). Table 3 further illustrates the results for the most informative muscle and fat IDPs associated with dynapenia and frailty, from our standardised logistic regression analysis.

**TABLE 3:** Standardised regression coefficients between dynapenia as well as frailty and the anthropometric covariate and the most informative muscle and fat IDPs.

	Models—dynapenia			Models—frailty	
	(1)	(3)		(9)	(11)
Age	1.527**	1.551**	Age	1.141**	1.143**
	(1.463, 1.594)	(1.487, 1.619)		(1.120, 1.163)	(1.122, 1.164)
Male	4.269**	3.740**	Male	1.027	1.148**
	(3.688, 4.941)	(3.262, 4.288)		(0.985, 1.068)	(1.106, 1.190)
Asian	1.377*	1.496*	Asian	1.477*	1.454*
	(1.026, 1.822)	(1.115, 1.976)		(1.280, 1.673)	(1.257, 1.650)
Black	0.607	0.680	Black	1.127	1.203
	(0.272, 1.163)	(0.305, 1.305)		(0.868, 1.386)	(0.944, 1.461)
Chinese	1.075	1.139	Chinese	1.236	1.170
	(0.554, 1.902)	(0.587, 2.016)		(0.867, 1.606)	(0.801, 1.540)
Others	0.892	0.935	Others	1.177	1.194
	(0.590, 1.299)	(0.619, 1.359)		(0.971, 1.383)	(0.989, 1.399)
Alcohol frequency [Daily]	0.681**	0.693**	Alcohol frequency [Daily]	0.522**	0.529**
	(0.601, 0.770)	(0.612, 0.784)		(0.450, 0.593)	(0.458, 0.601)
Alcohol frequency [1–4 times per week]	0.765**	0.774**	Alcohol frequency [1–4 times per week]	0.617**	0.620**
	(0.694, 0.844)	(0.703, 0.854)		(0.559, 0.674)	(0.563, 0.678)
Alcohol frequency [1–3 times per month]	0.780*	0.785*	Alcohol frequency [1–3 times per month]	0.790**	0.795**
	(0.679, 0.895)	(0.683, 0.901)		(0.714, 0.867)	(0.718, 0.871)
Smoking status [Previous]	0.966	0.950	Smoking status [Previous]	1.085*	1.101**
	(0.891, 1.046)	(0.877, 1.029)		(1.041, 1.129)	(1.057, 1.145)
Smoking status [Current]	0.996	0.949	Smoking status [Current]	1.202*	1.222*
	(0.794, 1.236)	(0.756, 1.177)		(1.087, 1.317)	(1.108, 1.337)
Townsend deprivation index	1.055*	1.054*	Townsend deprivation index	1.042*	1.043**
	(1.016, 1.094)	(1.016, 1.094)		(1.021, 1.063)	(1.022, 1.064)
MET	0.846**	0.849**	MET	0.908**	0.902**
	(0.813, 0.880)	(0.816, 0.884)		(0.887, 0.929)	(0.881, 0.923)
Total Muscle Volume	0.392**		Thigh IMAT Volume Index	1.396**	
	(0.361, 0.426)			(1.374, 1.418)	
Thigh Muscle Volume		0.416**	Mid-thigh IMAT/Muscle		1.408**
		(0.385, 0.449)			(1.383, 1.432)
			Not Frail|Pre Frail	1.104*	1.175**
				(1.102, 1.105)	(1.173, 1.177)
Constant	0.041**	0.044**	Pre Frail|Frail	70.934**	75.93**
	(0.037, 0.046)	(0.039, 0.049)		(68.062, 73.927)	(72.808, 79.186)
Observations	41,943	41,943	Observations	40,841	40,841
AIC	20854.5	20857.8	AIC	56672.4	56787.1

Values are presented as odds ratios (OR) and 95% confidence intervals (CI) in parentheses. * indicate statistically significant for p<0.05 , ** indicate statistically significant after Bonferroni correction ( $p=8.8{\times 10}^{-5}$ ). Models were adjusted for age, sex, ethnicity, alcohol intake frequency, smoking status, Townsend deprivation index, MET, as well as each muscle IDPs, separately. (1)—Total Muscle Volume; (3)—Thigh Muscle Volume; (9)—Thigh IMAT volume index; (11)—Mid-thigh IMAT/Muscle.

## 4 Discussion

In the present study we have muscle volumes and quality in a large prospective cohort of middle-aged and older adults in the UK Biobank. We have confirmed and expanded on previous studies looking at the impact of age, sex, and lifestyle factors on muscle strength and have identified muscle volume and quality measurements being strongly associated with dynapenia and frailty, respectively (Kalyani et al., 2014). Specifically, male participants exhibited higher muscle volumes compared to female participants, while when considering mid-thigh IMAT/muscle ratio and paraspinal muscle PDFF, female participants displayed higher values. Additionally, the prevalence of dynapenia and frailty was relatively low, consistent with the UK Biobanks generally healthy population. Both dynapenia and frailty were associated with increased age while they were negatively associated with alcohol and MET. Dynapenia was negatively associated with muscle volume and positively associated with mid-thigh IMAT/muscle and paraspinal PDFF levels. Muscle volume IDPs including total muscle and thigh measurements were more informative for dynapenia, while measures of muscle quality, specifically thigh IMAT volume index and mid-thigh IMAT/muscle ratio, were more informative for frailty, suggesting that different measurements may have distinct diagnostic values for various conditions.

Muscle volumes were significantly higher in male compared with female participants, even after correcting for height. We further show that total thigh IMAT content was significantly higher in male compared to female participants, however when presented as the mid-thigh IMAT/muscle ratio, this relationship was reversed, with higher levels of mid-thigh IMAT/muscle found in female participants. Similarly, paraspinal muscle PDFF, was also significantly higher in female participants. This clearly highlights the impact of the type of measurement on possible outcomes. Previously studies have reported higher whole-body and thigh IMAT levels in men (Gallagher et al., 2005; Sparks et al., 2021), but these were generally not corrected for muscle volume. Although, correcting for muscle mass negated reported sex differences in IMAT (Kim et al., 2017).

The prevalence of dynapenia (7.6%) and frailty (1.1%) were relatively low in our cohort, in agreement with reports that the UK Biobank cohort is a comparatively healthy population (Lyall et al., 2022). In this study, we defined dynapenia based on low muscle strength (Dodds et al., 2020; Wilkinson et al., 2022), without including reduced muscle mass/quality and/or reduced physical performance. Dynapenia was positively associated in men only in the model incorporating muscle volume measurements, while frailty showed a positive association in models that included total muscle, thigh muscle, iliopsoas muscle, mid-thigh IMAT/Muscle ratio and paraspinal muscle PDFF IDPs. Additionally, participants with both dynapenia and frailty demonstrated positive associations with age and lower physical activity level in all models, confirming earlier literature reports (Bouchard et al., 2009).

Our study further aimed to identify which of our measured muscle IDPs were associated with these health conditions, and which would potentially provide the best prognostic tool for identifying this population. We found that dynapenia was associated with reduced muscle volume and increased levels of mid-thigh IMAT/muscle and paraspinal PDFF. Notably, for dynapenia, the most informative muscle volume measurements were those with a greater proportion of body included such as total muscle or the entire thigh. On the other hand, for frailty markers of muscle quality, particularly thigh IMAT volume index to height squared and mid-thigh IMAT/Muscle were the most informative IDPs. This suggests that different IDPs may have different diagnostic values for different conditions. Interestingly, we observed that the muscle volumes indexed to height squared demonstrated a relatively poor fit in their associations with dynapenia.

A previous observational study, investigating the feasibility of using psoas or paraspinal muscles at the same axial slice as well as mid-thigh measurement to predict sarcopenia, found that the psoas muscle was more accurate than the paraspinal muscle, although inferior to mid-thigh measurement (Morrell et al., 2016). Additional studies in the UK biobank investigating associations between low muscle mass, malnutrition and sarcopenia with cancer (Kiss et al., 2023), found that in participants with obesity, only the BMI-adjusted muscle mass identified cases of low muscle mass and sarcopenia. They also reported that BMI-adjusted methods may be more suitable for determining these cases of malnutrition and sarcopenia; however, height-adjusted muscle mass showed a higher risk of these conditions occurring. Furthermore, in a previous study, Linge et al. (2020) reported that body size adjustments led to unnormalized correlations between muscle volume and body size. However, they also report that adjustments considering the distribution of height-adjusted measurements for each body size value significantly strengthened the associations between muscle volume and both functional and health outcomes. Future work using longitudinal data may provide better insights into which muscle volume and quality measurement can best associate with or even predict these health conditions.

Our study is not without limitations. The UK Biobank is a large cross-sectional study that is subject to selection bias with a “healthier” cohort than the wider UK population, excludes younger participants and potentially more severe clinical cases (Munafò et al., 2018). Furthermore, as the UK Biobank study includes a predominantly White population, future work is needed to further investigate imbalanced population data. Another potential limitation of this study is that although food intake such as energy and protein intake is widely used to assess the loss of muscle mass and function (Celis-Morales et al., 2018; Gedmantaite et al., 2020), these dietary parameters were not used in this study as they are reported 6.8 ± 1.5 years prior to the UK Biobank first imaging visit. Also, given the UK Biobank neck-to-knee acquisition, measurement of whole-body muscle volume could not be achieved, thus limiting direct comparisons with some literature values (Karlsson et al., 2015). We estimate that the current protocol lacks c. a. 9.75% of total muscle, corresponding to both arms and lower legs. However, as our findings were robust to the selection of muscle volume or quality measure, we consider it unlikely that results with a whole body measure would be dramatically different.

## 5 Conclusion

We present our muscle volume and quality IDPs derived from a large-scale population study. Our investigation revealed notable sex-related variations, with male participants exhibiting higher muscle volumes. In contrast, female participants displayed more significant mid-thigh IMAT to muscle ratio and paraspinal muscle PDFF. We further explored how the choice of specific muscle and fat measurements can impact the identification of diseases. Total muscle volume proved to be the most informative parameter for identifying dynapenia, whereas for frailty, muscle quality, especially thigh IMAT volume indexed to height squared, showed the strongest associations. Our research also highlights that the choice of muscle measurements can significantly affect the assessment of disease. However, it is essential to note that most of these measurements consistently reveal differences related to age, sex, and clinical conditions, emphasising their value in assessing muscular health. Our findings underscore the significance of these muscle metrics in evaluating the impact of various factors on muscular health within diverse populations. These metrics can potentially serve as valuable tools for future research and clinical applications.

## Data Availability

The UK Biobank resource is available to bona fide researchers for health-related research in the public interest. All researchers who wish to access the research resource must register with UK Biobank by completing the registration form in the Access Management System (AMS - https://bbams.ndph.ox.ac.uk/ams/).
